# Osteogenic and bactericidal surfaces from hydrothermal titania nanowires on titanium substrates

**DOI:** 10.1038/srep36857

**Published:** 2016-11-18

**Authors:** P. M. Tsimbouri, L. Fisher, N. Holloway, T. Sjostrom, A. H. Nobbs, R. M. D Meek, B. Su, M. J. Dalby

**Affiliations:** 1 Centre for Cell Engineering, University of Glasgow, Glasgow, Scotland, UK; 2 School of Oral and Dental Sciences, University of Bristol, Bristol, UK; 3 Golden Jubilee National Hospital, Clydebank, Glasgow, Scotland, UK; 4 Department of Orthopaedics, The Queen Elizabeth University Hospital, Glasgow, Scotland, UK

## Abstract

Nanotopographical cues on Ti have been shown to elicit different cell responses such as cell differentiation and selective growth. Bone remodelling is a constant process requiring specific cues for optimal bone growth and implant fixation. Moreover, biofilm formation and the resulting infection on surgical implants is a major issue. Our aim is to identify nanopatterns on Ti surfaces that would be optimal for both bone remodelling and for reducing risk of bacterial infection. Primary human osteoblast/osteoclast co-cultures were seeded onto Ti substrates with TiO_2_ nanowires grown under alkaline conditions at 240 °C for different times (2, 2.5 or 3 h). Cell growth and behaviour was assessed by scanning electron microscopy (SEM), immunofluorescence microscopy, histochemistry and quantitative RT-PCR methods. Bacterial colonisation of the nanowire surfaces was also assessed by confocal microscopy and SEM. From the three surfaces tested the 2 h nanowire surface supported osteoblast and to a lesser extent osteoclast growth and differentiation. At the same time bacterial viability was reduced. Hence the 2 h surface provided optimal bone remodeling *in vitro* conditions while reducing infection risk, making it a favourable candidate for future implant surfaces.

Preventing biofilm formation on surgical implants is a major challenge in the biomaterials and medical devices field. Infection can result in traumatic and costly revision surgery^1^,^2^,^3^, and the prevention of such infection, without impeding the function of the implant, is of significant importance. Currently, only very few studies have assessed bacterial and osteoblast adhesion to potential implant surfaces suggesting that differential effects can be obtained^4^,^5^. Most studies remain focussed on differentiation of bone forming cells such as mesenchymal stem cells (MSCs) or osteoblasts on biomaterial surfaces.

Indeed, previous work using nanotopography to study mesenchymal stem cell (MSC) differentiation has shown that MSCs can be targeted to produce tissue specific lineages such as osteoblasts^6^,^7^,^8^. A limitation of these approaches is that only one cell type is used and this is not indicative of the more complex *in vivo* situation. *In vivo,* osteoblasts and osteoclasts work together to maintain bone homeostasis with osteoblasts forming new bone and osteoclasts removing old bone. Osteoblasts have a regulatory role in osteoclastogenesis through their ability to secrete key cytokines and growth factors for the formation and activity of osteoclasts (Supplementary Figure 1). The first response factors are macrophage-colony stimulating factor (M-CSF) and receptor activator of nuclear factor κB ligand (RANKL) that induce osteoclastogenesis by fusion of macrophages^9^,^10^,^11^. This is followed by the expression of tartrate resistant acid phosphatase (TRAP), cathepsin K and osteoclast associated receptor (OSCAR) from the newly formed osteoclasts. Conversely, osteoblasts can inhibit the process through expression of osteoprotegrin (OPG)^12^.

Over recent years, there have been attempts to build complexity into *in vitro* testing. For example, osteoblast and monocyte or peripheral blood mononuclear cell (PBMC) co-cultures in combination with M-CSF and RANKL have been reported^13^,^14^,^15^. Further studies reported the use of porcine co-cultures of bone marrow stromal cells (BMSCs) and bone marrow haematopoetic cells (BMHCs)^16^, or of human co-cultures of BMSC and CD34 + BMHC^17^ or MSC and PBMCs^18^. We recently developed a methodology to allow mesenchymal bone marrow stromal cell (BMSC)/bone marrow haematopoetic cell (BMHC) co-cultures that could form mature osteoblasts and osteoclasts in culture in response to materials^19^,^20^. Further, we have illustrated that nanoscale topography in polymers^19^ and in titanium can present positive osteogenic cues while osteoclastogenesis remains unchanged; i.e. specific bioactivity has been indicated^21^. A more ideal i*n vitro* test for candidate materials would focus both on understanding osteoblast/osteoclast growth and differentiation and also would understand if surfaces are resistant to infection.

Ti and its alloys are commonly used materials for applications including dental and orthopaedic implants^22^,^23^. Antimicrobial Ti surfaces have been achieved with binding of antibiotics^24^, antimicrobial peptides^3^ or nanoparticles which have bactericidal effects^1^. Further, recent work illustrated that high aspect ratio topographies, designed using carbon black, can be bactericidal^25^ and more recently on Ti^26^. Bringing together the potential of specific bioactivity and bactericidal effects that topography can have would be a powerful approach in orthopaedic bioengineering without the need for further coatings or drug agents.

Here we employ our *in vitro* co-culture system on high aspect ratio (average height of 1 μm and approximately 25 nm in diameter) TiO_2_ nanowires grown on Ti substrates. We show that the nanowire TiO_2_ substrates support growth of osteogenic cells without stimulating a further associated osteoclast response. At the same time bacterial viability of a pathogen (Pseudomonas Aeruginosa) commonly involved to medical device-associated infections^27^,^28^, is reduced on the optimal nanotopography for bone marrow (BM) cell growth, providing a good candidate surface for new orthopaedic and dental implants.

## Results

### Nanowire TiO_2_ surfaces

**Figure 1:**
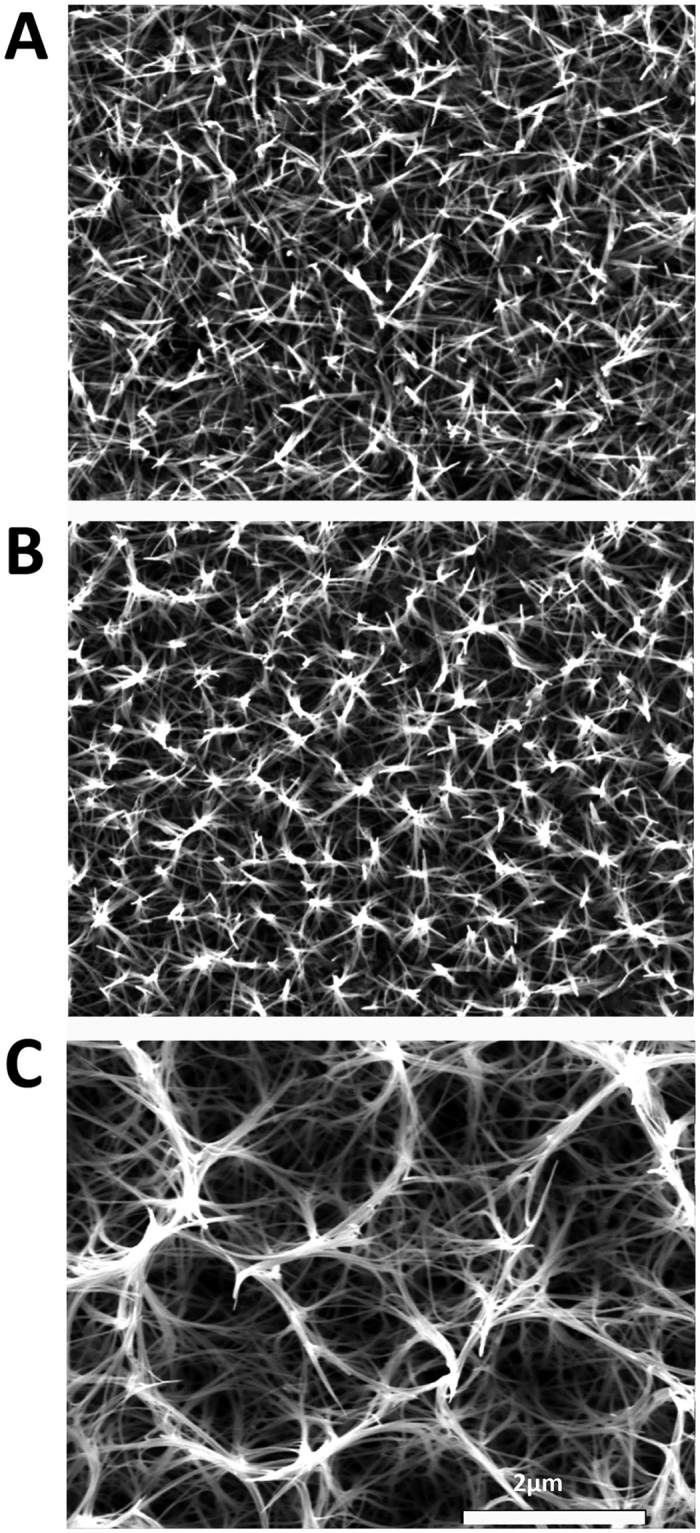
SEM images of Ti nanosurfaces. Hydrothermal treatment of titania (TiO_2_) surfaces changed the texture of the surface from a homogeneously dense coverage of spike-like structures at 2 h (**A**) or 2.5 h (**B**) with an average height of 1 μm to longer 2 μm structures that slightly intertwined at their tips, adopting small pocket-like arrangements at 3 h (**C**).

The hydrothermal TiO_2_ nanowire synthesis process was previously described but in this study we have focused on shorter synthesis times (2–3 h). The nanowire titanium surfaces were generated by the application of an alkaline hydrothermal process as a function of time i.e. 2 h, 2.5 h or 3 h respectively. This method made the modification of titanium surfaces with controllable dimensions of TiO_2_ nanowires possible. The varying time for hydrothermal treatment (240 °C) generated a layer of homogeneously packed, spike-like structures with an average height of ca. 1 μm and diameters of approximately 25.1 nm ± 2.7 nm (Fig. 1A–C) and (Supplementary Figure 2). Increasing treatment times from 2 to 3 h gave rise to slight variations in the wire arrangements from more compact brush-like spiky features at 2 h to a slightly intertwined structure at 3 h forming small bundles (co-allescence) with width 222 nm ± 53.8 as the nanowires grew in height by approx. 1 μm with increased process time. The nanowire diameter remained unchanged with increased process time.

### BM co-cultures on TiO_2_ nanowires

**Figure 2:**
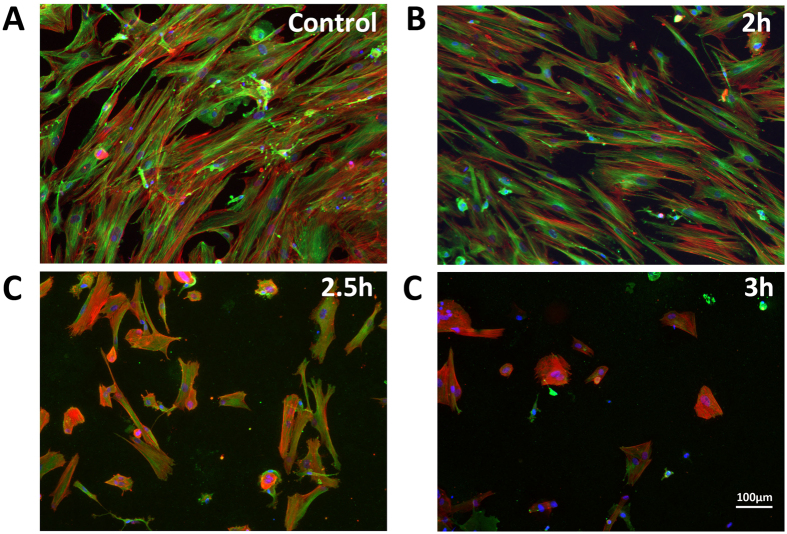
Immunofluorescence micrographs indicating cell morphology and spread on the different nanowire surfaces. On (**A**) the polished control surfaces cells are well spread with a uniform distribution and intense tubulin staining. A similar appearance is adopted on (**B**) the 2 h surfaces. As the surface complexity increases, the cell spread and numbers diminish on the (**C**) 2.5 h and (**D**) 3 h surfaces. Red: actin, Green: tubulin, Blue: nucleus. Scale bar as indicated.

BMSC/BMHC co-cultures, were cultured on 14 mm diameter polished control and 2 h, 2.5 h or 3 h nanowire surface Ti discs. Initially cells were cultured for one week to determine cell growth and behaviour on the surfaces. Cells were well spread with well organised cytoskeleton on the polished, control surfaces with the cells approaching confluence (Fig. 2A–D). A similar appearance and behaviour was observed on the 2 h nanowires although with slightly less dense cell coverage. However, as the topography changed with treatment time, on the 2.5 h and 3 h nanowires the cells grew in clusters, appeared smaller, less spread and reduced in numbers with tubuln staining becoming more dense around the nucleus and less organised in the cytoplasm.

**Figure 3:**
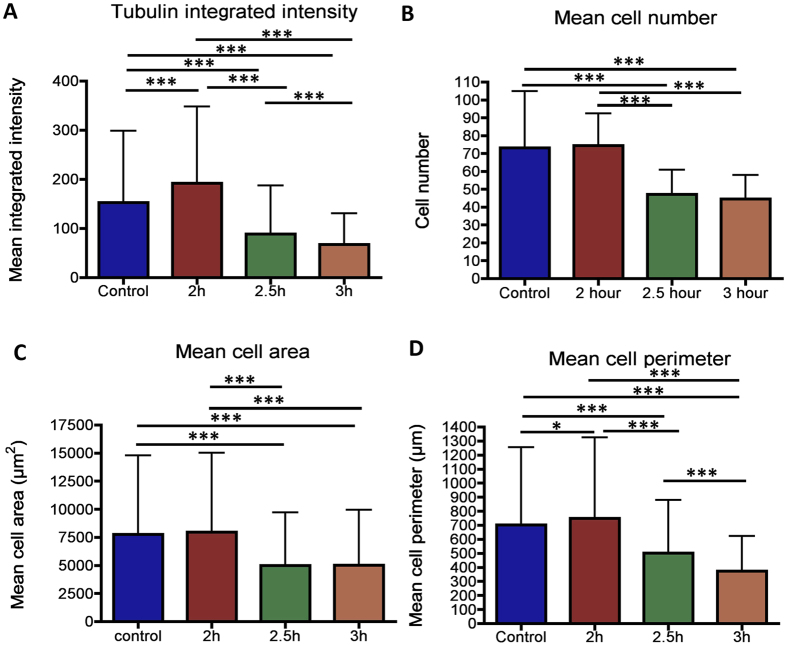
Graphical representation of cell number and size distribution on the nanowires. Reduced mean intensity in interwined versus smoother surfaces (**A**). Fewer cells (**B**) and less spread cells (**C,D**) were observed as surface complexity increased in comparison to the control. Cellprofiler was used to obtain cell descriptors and cell numbers. Comparison was by ANOVA *P < 0.05, ***P < 0.001, n = 30 images.

Quantitative analysis of the fluorescence images using Cellprofiler showed that there was a significant decrease in the tubulin mean intensity in the more interwined 2.5 and 3 h surfaces than the control. The cells on the 2 h surface displayed a comparable tubulin expression level to the control (Fig. 3A). Furthermore, cell numbers present on the more interwined surfaces were greatly reduced in comparison to the control and 2 h surface (p < 0.001) (Fig. 3B). Cell area (Fig. 3C) and perimeter (Fig. 3D) were also significantly reduced for the more interwined 2.5 h or 3 h surfaces compared to 2 h or control surfaces (p < 0.001, for both parameters), indicating that the cells prefer more compact or smoother surfaces to adhere and spread. See Supplementary Figure 3 for SEM detail of cell/topographical interactions.

These results indicated that, from the three surfaces tested, the 2 h nanowires were the preferred surface. Subsequent studies therefore concentrated on this topography.

### Cell differentiation

#### Gene expression

A number of genes were tested by qRT-PCR in order to assess the effects elicited by the 2 h nanowire surface on osteoblast/osteoclast differentiation in comparison to the polished control. Gene expression was assessed at 14 days (D14) and at 35 days (D35) to ensure that any time-related expression differences were not missed.

**Figure 4:**
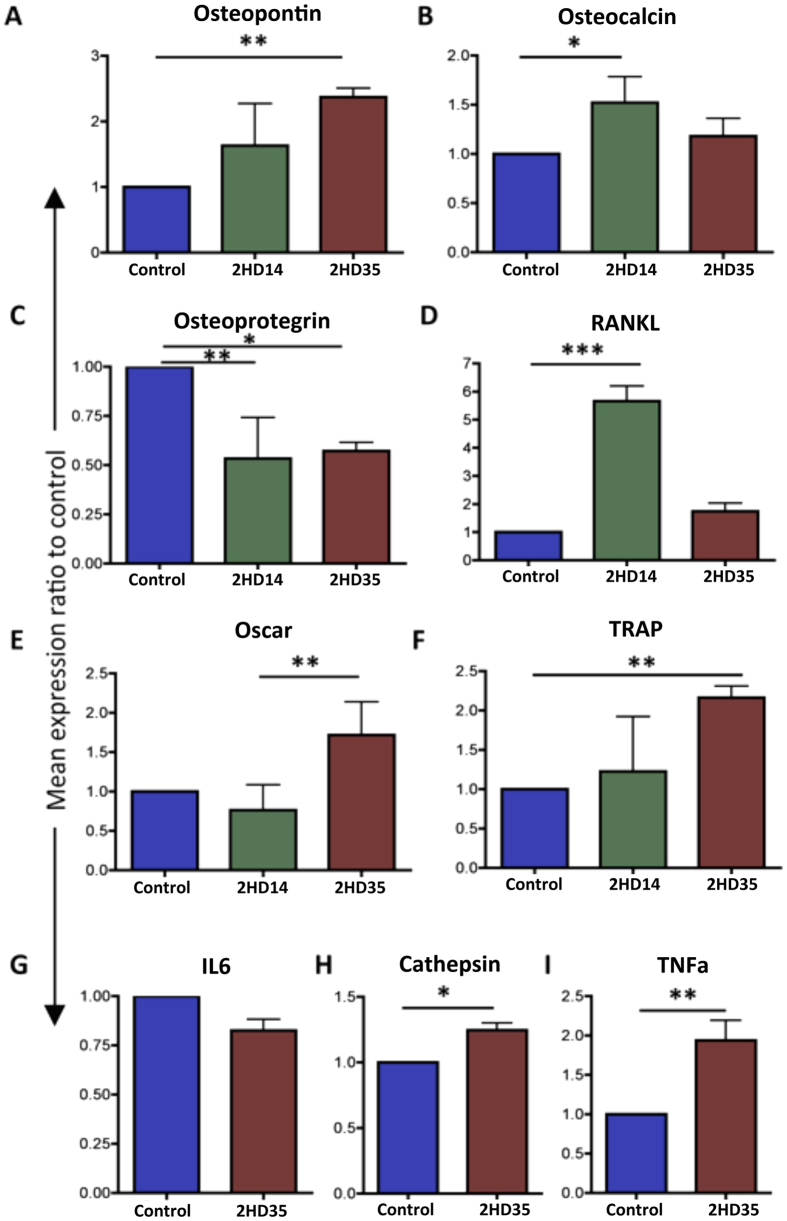
Mean gene expression in co-cultures on 2 h nanowire Ti substrate in comparison to the polished Ti control at days 14 and 35. (**A**) Osteopontin, (**B**) Osteocalcin, (**C**) Osteoprotegrin, (**D**) RANKL, (**E**) OSCAR, (**F**) TRAP, (**G**) Interleukin 6 (IL6), (**H**) Cathepsin and (**I**) Tumour necrosis factor a (TNFa). Results are shown as ratio of control. n = 3. Comparison was by ANOVA *p < 0.05, **p < 0.01, ***p < 0.001).

A significant increase in expression of the osteogenic lineage genes OPN and OCN was observed on the 2 h nanowire surface in comparison to the polished control (Fig. 4A,B, p < 0.01 and p < 0.05 respectively). The expression of OPG, a negative regulator of osteoclastogenesis, was found to be significantly (p < 0.05) downregulated in co-cultures on the 2 h nanowires in comparison to the control (Fig. 4C). At the same time, the expression of the osteoclastogenesis inducer RANKL was found to be increased on the 2 h nanowires (p < 0.001) at d14 but reduced later (Fig. 4D). Concomitantly, the expression of osteoclast-associated receptor OSCAR (p < 0.01) (Fig. 4E), TRAP (p < 0.01) (Fig. 4F), cathepsin (p < 0.05) (Fig. 4H) and inflammation related gene TNFα (p < 0.01) (Fig. 4I) was significantly increased in comparison to the control. Expression of the cytokine IL-6 was found to be slightly reduced on the 2 h nanowires when compared to the control (Fig. 4G) in the co-cultures. IL-6 is involved in bone remodelling and can have positive effects on both osteoblastogenesis and osteoclastogenesis^29^,^30^.

#### Osteoblast differentiation

**Figure 5:**
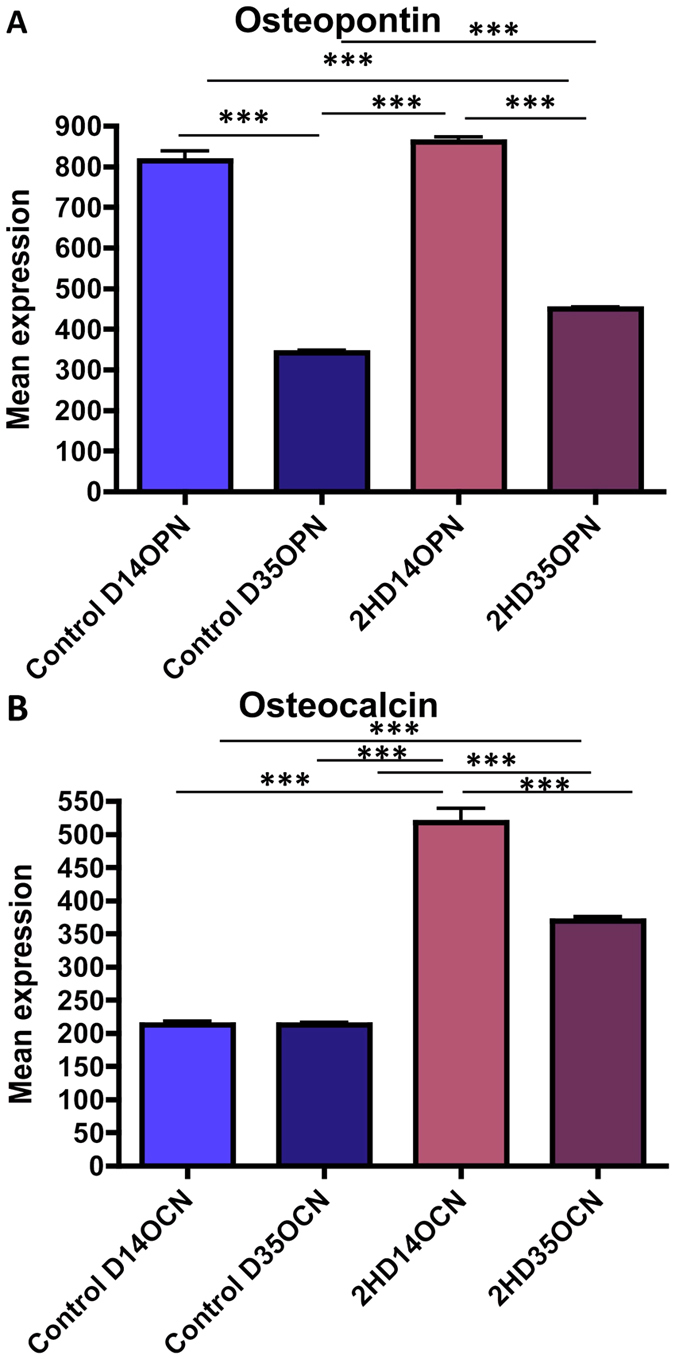
Immunofluorescence results for bone related markers. (**A**) Osteopontin mean intensity was higher at D14. (**B**) Osteocalcin mean intensity was higher on the 2 h surface in comparison to the control at both time points. Statistics by ANOVA ***p < 0.001. n = 30 images.

In order to further assess the osteogenic properties of the 2 h nanowires as a potential candidate for implant surface application, the protein-level expression of OPN and OCN was assessed (Fig. 5A). Expression of OPN expression was was seen to maximal at D14 with similar exression levels in MSCs on control and on 2 h nanowires. However, expression on the 2 h wires was significantly higher than control at D35 (p < 0.001). OCN expression was, again, maximally expressed at D14, but was significantly higher on the 2 h nanowire surface in comparison to the control cultures at both time points (p < 0.001) (Fig. 5B).

#### Osteoclastogenesis

**Figure 6:**
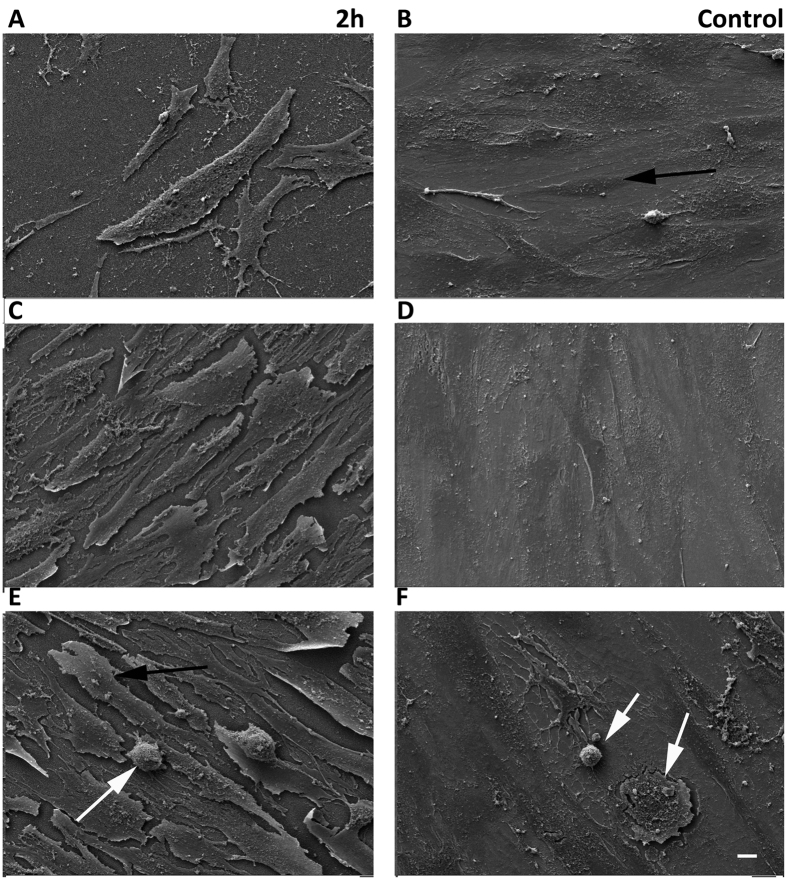
SEM images of 2 h Ti substrate at D3 (**A,B**), D14 (**C,D**) and D35 (**E,F**) at 1000x magnification osteoclast progenitors (white arrow) and osteoblasts (dark arrow) are shown. Images provide evidence of co-culture of osteoblasts and osteoclast progenitors, with a significant decrease in osteoclast progenitor cells on the 2 h nanowire surface versus the control substrate. Image F shows osteoclast progenitor cells on the control substrate at day 35. A less mature osteoclast progenitor cell is seen in image E at day 35, and is seen to be adherent to an osteoblast cell, not to the nanowire substrate surface. Scale bar 100 μm.

**Figure 7:**
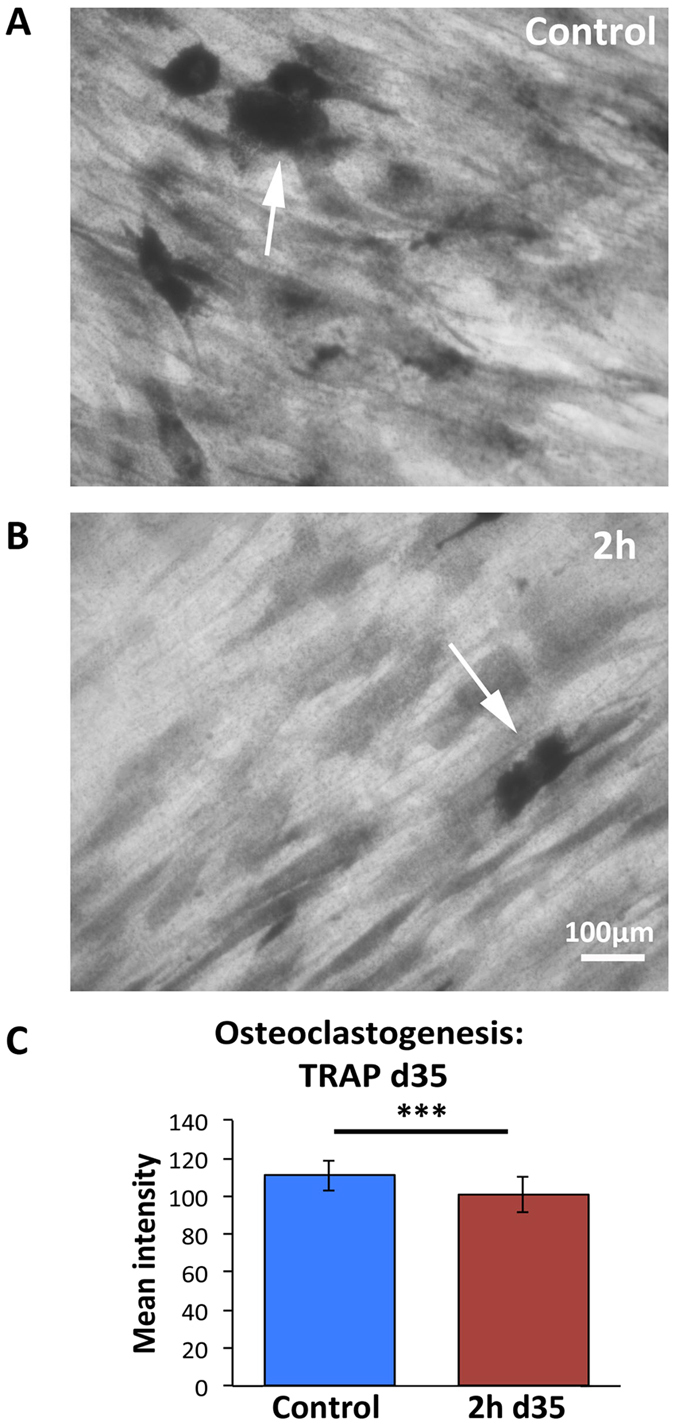
TRAP histochemical images. (**A**) Control and (**B**) 2 h nanowire substrate respectively at 20x magnification. On the control substrate, TRAP positive macrophages or pre-osteoclasts indicative of osteoclastic activity can be seen (arrow). Very few TRAP positive macrophages were present on the 2 h nanowire substrate. (**C**) TRAP staining mean intensity was significantly lower on the 2 h nanowire surface in comparison to the control as measured by an unpaired T-test, ***P < 0.001.

SEM imaging showed very few multinucleated osteoclast–like cell were noted at any time point (D3, 14 and 35) indicating that the BMHCs were not fusing to form mature osteoclasts (Fig. 6). Further, histochemical TRAP staining was performed and confirmed the SEM observation. Very low numbers of TRAP reactive cells were noted overall (Fig. 7A,B) and densitometry measurements indicated that less TRAP reactive areas were stained in the co-culture on the 2 h nanowire substrates in comparison to the polished control (Fig. 7C). A representative mature osteoclast is shown in Supplementary Figure 4.

### Bactericidal properties of 2 h nanowires

**Figure 8:**
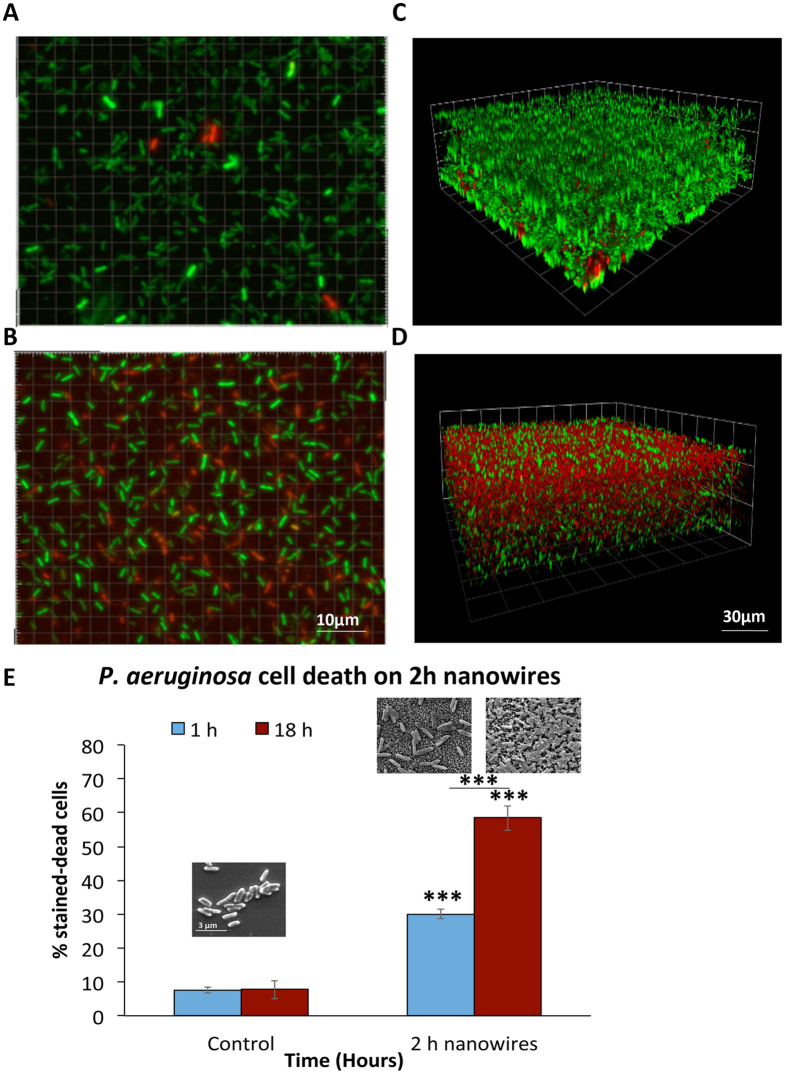
Attachment of P. aeruginosa on control (polished Ti surfaces) and 2 h nanowire surfaces (**A,B** respectively) Bacterial cells were stained with Live/Dead BacLight, where cells with intact membranes stain green (SYTO 9) and cells with damaged membranes stain red (propidium iodide). Confocal images of P. aeruginosa biofilm, after 18 h incubation, attached to control (polished titanium) (**C**) and 2 h nanowires (**D**). Bacterial cells appear to have been punctured by the nanowires, resulting in collapsed cells (black arrow) (**E**) Average percentage of stained-dead P. aeruginosa cells on control and 2 h nanowire surfaces. Insets of SEM images reveal more pierced looking cells upon 18 h attachment compared to 1 h, confirming live/dead results (58% kill). Results analysed using an unpaired T-test, ***P < 0.001. Scale as shown.

Further to the osteogenic potential of the 2 h nanowires we wanted to examine the bactericidal properties of this topography. Figure 8A,B show representative fluorescence micrographs of attachment of *P. aeruginosa* to control (polished Ti surfaces) and 2 h nanowire surfaces respectively at 1 h. Figure 8E shows the average percentage of stained-dead *P*. *aeruginosa* cells on control and 2 h nanowire surfaces at 1 h and 18 h bacterial cultures. The 2 h nanowires displayed a ~30% killing efficacy after 1 h and 58% killing efficacy after 18 h of bacterial culture. This can be clearly seen in Fig. 8C,D of confocal images and the insets in Fig. 8E of *P. aeruginosa* attached to 2 h and polished Ti surfaces. Cells on the polished Ti looked healthy whereas cells on the 2 h exposed surfaces appeared to have been punctured by the nanowires at 1 h culture and even more so after 18 h of culture. Collapse of the cell and spreading between the nanowire structures is evident.

## Discussion

Material surface modulations at the nanoscale have been shown to affect cell adhesion, morphology and phenotype^6^,^8^,^31^,^32^,^33^. Hence, permanent surface topographies which can be tailored to selectively support the desired biological activity over long periods of time are advantageous for implant technologies^34^. Biomimicking natural antimicrobial surfaces (e.g. insect wings) appears to support this rationale with clear preference for physico-mechanical mechanisms of action, independent of chemistry^35^,^36^,^37^. In this regard, different types of TiO_2_ nanowires were hydrothermally grown on Ti surfaces at 2 h, 2.5 h and 3 h and their effects on cell growth, phenotype and their potential use in bone remodeling were assessed in this study.

We recently developed osteoblast/osteoclast progenitor co-cultures where mature phenotypes of both progenitor lines could develop on polymeric^6^,^19^ and metallic^21^ nanostructures. In this study we have used the same co-culture system on the new, hydrothermally synthesised, surfaces. From the three topographies assessed (2 h, 2.5 h and 3 h), only the 2 h nanowires were permissive for normal cell morphology. In addition, qualitative and quantitative analysis of osteogenic markers OPN and OCN at the transcript and protein level demonstrated an increase in osteogenesis on the 2 h nanowire substrate.

We were motivated to understand if topographical bioactivity was specific (osteoblast only) or general (osteoblast and osteoclast promoting). Ideally, osteogenesis would be increased while osteoclastogenesis remained normal^12^,^16^. Assessment of the effects of the 2 h nanowire surface on osteoclastogenesis via gene expression (RANKL, OSCAR, TRAP, cathepsin) appeared to indicate that the wires may induce osteoclast formation. However, morphological and protein-level phenotypical observation (SEM and TRAP staining) indicated little difference, even a potential decrease in osteoclast activity over the 35 day culture time. Potentially, a combination of possible post-translational modifications and inhibitory signals elicited by the nanowire surface may explain the lack of mature osteoclasts as shown by SEM and TRAP staining. The results suggest that the 2 h TiO_2_ nanowire surface is specifically bioactive.

Furthermore, similarly to the recent report by the Ivanova group^26^ the 2 h nanowires were highly bactericidal towards the Gram-negative and motile bacterium *P. aeruginosa*, as shown by SEM imaging and viability assay with a 30% kill rate after 1 h and 58% after 18 h in culture. The antibacterial properties exhibited here by the 2 h nanowires against *P. aeruginosa* could be crucial *in vivo* by inhibiting early stage bacterial adhesion and thus improving the body’s ability to fight infections. This is clearly important as increased bone contact without increased resorption, combined with reduced risk of infection, would be the optimal implant surface. A major concern for implant technologies is infection as rapid failure of implants can result^28^,^38^,^39^. This worry is in tune with world-wide concerns of growing antibiotic resistance and even the possibility of the post-antibiotic era. The development of antibiotics has been vital to our survival, yet antimicrobial resistance is threatening to make them ineffective. WHO estimates that antibiotic treatments add an average of 20 years to all of our lives^40^. However, overuse of antibiotics has put pressure on bacteria to evolve resistance and this is leading to a potential increase in scenarios where implants cannot be used due to fear of infection. It is thus clear that development of innovative methods, such as implants that have innate antimicrobial properties, is a priority. However, this can’t be at the sacrifice of desired effects such as osseointegration.

Assessment of *in vitro* co-culture has demonstrated the osteogenic properties of 2 h TiO_2_ nanowires grown on Ti substrate without an unwanted osteoclastic response. Hence, we have a successful co-culture approach on titanium surfaces providing an improved *in vitro* analysis of the implant-bone environment. Furthermore, the bactericidal activity of 2 h nanowires against *P. aeruginosa* could result in reduced risk of infection and subsequent long-term complications and thus facilitate tissue integration without using antibiotics. However, more bacterial strains, including some common orthopaedic pathogens (e.g. Gram-positive *Staphlylococcus aureus*), should be tested to fully characterise the bactericidal efficacy of such surfaces. Finally, as these surfaces are readily manufactured using a simple process, they can be easily grown onto different titanium implants of complex shape and size.

## Materials Methods

### Generating titania nanowires on Ti substrates

The Ti disk samples were prepared from a 0.9 mm thick ASTM grade 1 Ti sheet (Ti metals Ltd, UK). They were polished to a mirror image and ultrasonically cleaned in water and ethanol. Nanowires were created by immersing the Ti disks in 1 M NaOH in a PTFE lined steel vessel (Acid Digestion vessel 4748, Parr Instrument Company, USA), at a temperature of 240 °C for 2, 2.5 or 3 h. The vessel was removed after each time point from the oven and allowed to cool to room temperature. The samples were rinsed in water and ethanol, sequentially and they were subsequently heat treated at 300 °C for 1 h prior to ion exchange in HCl. To convert the sodium titanate nanowires to TiO_2_ the samples were immersed in 0.6 M HCl for 1 h, rinsed in water and ethanol, and finally heat treated at 600 °C for 2 h.

### Human BMSC and human BMHC isolation

BM was obtained from hip and knee arthroplasty surgeries from three healthy patients after patient informed and signed consent. This residual tissue for cell isolation and subsequent cell use described in this manuscript was approved by the National Health Service Research Ethics Comitee (NHS REC) with approval ref number 04/S0702/22. All methods were performed in accordance with the relevant guidelines and regulations approved by NHS REC. The BM aspirate was split into two vials and washed twice in basal medium (DMEM (Sigma) supplemented with 10% foetal bovine serum, 200 mM L-glutamine (Invitrogen), 100 mM sodium pyruvate, 1% MEM NEAA (Gibco) and antibiotics (6.74 U/ml Penicillin-Streptomycin, 0.2 μg/ml Fungizone)) and each time centrifuged at 376 g for 10 min. The cell pellets were resuspended in basal medium and overlaid on a Ficoll layer. This was centrifuged at 445 g for 45 min. The mononuclear interface layer was transferred in a new vial and further washed twice in basal media. The cells were transferred to an appropriately sized cell culture flask and incubated at 37 °C with 5% humidified CO_2_. At day 3, post selection, the non-adherent cells were transferred to a new flask and cultured separately as bone marrow hematopoetic cells (BMHC). The remaining adherent cells were cultured for a further 7–10 days until a confluent bone marrow stromal cells (BMSC) layer was identified.

### Co-cultures on titania nanowire surfaces

After 7 days of culture, the BMSCs were trypsinised and resuspended in basal media at a density of 1 × 10^4^ cells/ml/Ti substrate. Five days later, 1 ml of BMHC suspension was added at a concentration of 1.5 × 10^5^ cells/ml to form the co-culture. Medium was replaced every 3 days of the co-culture.

### Histochemistry: TRAP staining

At day 35 of co-culture, histochemical analysis was performed on duplicate 2 h, 2.5 h and 3 h nanowire or polished control substrates to assess osteoclastogenesis. Samples were fixed in 4% formaldehyde fixative for 30 s, stained with TRAP (Acid Phosphatase Leukocyte No.387, Sigma-Aldrich) according to the kits instructions and counterstained for 10 min in haematoxylin solution followed by a wash in H_2_O.

### Immunofluorescence

At day 35 of co-cultures, 2 discs of each substrate were analysed by immunofluorescence staining as described previously in ref. 8. Briefly, cells were fixed in 4% formaldehyde fixative at 37 °C for 15 min and permeabilized at 4 °C for 5 min. The samples were blocked with 1% BSA/PBS at 37 °C for 5 min and stained with the appropriate primary antibodies for osteopontin (OPN), osteocalcin (OCN) or Tubulin (1:50 in 1% BSA/PBS, Autogen Bioclear). Actin fillaments were stained with phalloidin (1:100, Invitrogen). Two washes in 1 × PBS/0.5% Tween-20 (3 × 5 min at room temperature) followed and a secondary, biotin-conjugated antibody (1:50, horse monoclonal anti-mouse (IgG), Vector Laboratories) was added for 1 h at 37 °C. The samples were washed and streptavidin was added (1:50, Vector Laboratories) at 4 °C for 30 min, before the samples were given a final wash and mounted in Vectashield mounting medium (Vector Laboratories) containing DAPI to stain the nucleus. Visualisation was via a fluorescence microscope (Zeiss Axiovert 200 M, 10x magnification, NA 0.5). Files generated in JPEG or TIFF format. Comparisons of staining intensity between substrates was analysed by Image J software version 1.42q.

### Scanning Electron Microscopy (SEM)

SEM was performed at days 3, 14 and 35 of co-culture on duplicate 2 h, 2.5 h and 3 h nanowire or polished control Ti substrates. The samples were fixed in 4% gluteraldehyde for 1 h at 4 °C and then washed in 0.2 M sodium cacodylate (pH 7.4). They were post fixed in 1% osmium tetroxide and washed once in sodium cacodylate. The samples were then immersed in a 1% (v/v) tannic acid/0.1 M sodium cacodylate solution for 1 h and then washed in 0.2 M sodium cacodylate before the dehydration steps through an incremental (70–100% v/v) alcohol series. A hexamethyldisiloxane step was conducted prior to sputter coating (20 nm gold/palladium). A Zeiss Sigma FE-SEM microscope was used for sample visualisation.

### qRT-PCR

Total RNA was extracted from day 14 and 35 time point cultures using a Qiagen RNeasy Micro kit and protocol. To standardise for quantitative analysis, equal amounts of RNA from each sample were used for cDNA synthesis using the Qiagen QuantiTect RT-PCR kit and protocol. qRT-PCR was carried out using the Qiagen Quantifast SYBR Green kit and the reactions run in the 7500 Real Time PCR cycler from Applied Biosystems. Three biological (and 2 technical replicates from each) replicates were tested at each time point. Expression of test genes (alkaline phosphatase, OPN, OCN, SOX9, OPG, OSCAR, RANKL, interleukin 6 (IL-6), TRAP, tumour necrosis factor α (TNFα) and cathepsin-K) was normalised against house keeping gene GapDH. The primer sequences (Table 1) for the genes were validated by dissociation curve/melt curve analysis.

### Bacterial culture preparation

*Pseudomonas aeruginosa* ATCC 27853 was grown overnight in 10 ml Luria Bertani (LB) broth in a 37 °C shaker incubator at 220 rpm. The bacterial suspension was then sub-cultured into fresh LB broth to OD_600_ 0.1 and further incubated until mid-exponential phase was reached (OD_600_ 0.5–0.6). Bacterial cells were then harvested (7 min, 5000 *g*) and washed three times in Tris-HCl buffer (pH 7, Sigma). Bacterial cells were finally re-suspended in Tris-HCl buffer to OD_600_ 0.3 (approx. 10^7^ CFU/ml).

### Bacterial adhesion assay

Sterile 2 h nanowire discs and control discs (polished Ti) were placed into a 12-well microtitre plate and incubated with 2 ml bacterial suspension for 1 h or 18 h at 37 °C under static conditions. Plates were placed into a plastic container containing moist tissues to maintain humidity. After incubation, discs were rinsed 3 times in Tris-HCl buffer to remove non-adherent bacteria, and submerged in 1 ml Live/Dead^®^ *Bac*Light™ bacterial viability stain (Invitrogen, as per manufacturers’ instructions) for 15 min at room temperature. Discs were then rinsed twice in Tris-HCl buffer, and submerged in 1 ml Tris-HCl buffer for visualisation by confocal microscopy. (Multilaser CLSM Leica SP511). Five Z-stack images per surface were taken and Volocity 6.3 cellular imaging and analysis software was used to count the number of cells with intact membranes (SYTO 9, green) and the number of cells with damaged membranes (propidium iodide, red). The average percentage of damaged cells was determined by dividing the damaged cells by the total number of cells on the surface (*100). All experiments were carried out in triplicate and repeated on separate occasions.

### Scanning Electron Microscopy (SEM) of bacterial cultures

Surfaces attached with *P. aeruginosa* were fixed by immersion in a 2.5% gluteraldehyde solution (Sigma Aldrich) dissolved in 0.1 M potassium phosphate buffer (Sigma Aldrich) for 2 h at room temperature. An alcohol dehydration stage was then performed by immersing the surfaces in 20%, 40%, 60%, 80% and 100% ethanol (Sigma Aldrich) for 10 mins each prior to 10 mins in hexamethyldisilazane (Sigma Aldrich). Surfaces were then air dried, mounted onto carbon stubs and sputtered with platinum for 30 seconds. A Quanta 400FEI Scanning Electron Microscope (SEM) was used to visualise the surfaces.

## Additional Information

**How to cite this article**: Tsimbouri, P. M. *et al*. Osteogenic and bactericidal surfaces from hydrothermal titania nanowires on titanium substrates. *Sci. Rep.* **6**, 36857; doi: 10.1038/srep36857 (2016).

**Publisher’s note:** Springer Nature remains neutral with regard to jurisdictional claims in published maps and institutional affiliations.

## Supplementary Material

Supplementary Information

## Figures and Tables

**Table 1:** PCR primer sequences.

Target Gene	Forward Sequence	Reverse Sequence
OSCAR	CCAGCTCTAGCGGGTATCTG	GACGGAGTGATGTCTGTGTGAC
OPG	GAAGGGCGCTACCTTGAGAT	GCAAACTGTATTTCGCTCTGG
RANKL	TGATTCATGTAGGAGAATTAAACAGG	GATGTGCTGTGATCCAACGA
IL-6	GATGAGTACAAAAGTCCTGATCCA	CTGCAGCCACTGGTTCTGT
TRAP	GGACTGAAGGGACTCCTGAAT	GGTCCCTGAGCCTTTATTCC
TNFα	CAGCCTCTTCTCCTTCCTGAT	GCCAGAGGGCTGATTAGAGA
Cathepsin-K	GCCAGACAACAGATTTCCATC	CAGAGCAAAGCTCACCACAG
Alkaline phosphatase	AGAACCCCAAAGGCTTCTTC	CTTGGCTTTTCCTTCATGGT
GapDH	GTCAGTGGTGGACCTGACCT	ACCTGGTGCTCAGTGTAGCC
OPN	AGCTGGATGACCAGAGTGCT	TGAAATTCATGGCTGTGGAA
OCN	CAGCGAGGTAGTGAAGAGACC	TCTGGAGTTTATTTGGGAGCAG
