# Supplementary material for: Osteogenic and bactericidal surfaces from hydrothermal titania nanowires on titanium substrates
Source: Sci Rep. 2016 Nov 18;6:36857. doi: 10.1038/srep36857 (PMC5114696; doi:10.1038/srep36857)
Supplement: Supplementary Information [file srep36857-s1.pdf]

Osteogenic and bactericidal surfaces from hydrothermal titania nanowires on titanium substrates

P.M. Tsimbouri[1*], L. Fisher[2], N. Holloway[3] ,T. Sjostrom[2], A.H. Nobbs[2], R.M.D Meek[4], B. Su[2], M.J. Dalby[1]

1 Centre for Cell Engineering, University of Glasgow, Glasgow, Scotland, UK

2 School of Oral and Dental Sciences, University of Bristol, Bristol, UK

3 Golden Jubilee National Hospital, Clydebank

4 Department of Orthopaedics, The Queen Elizabeth University Hospital, Glasgow, Scotland, UK

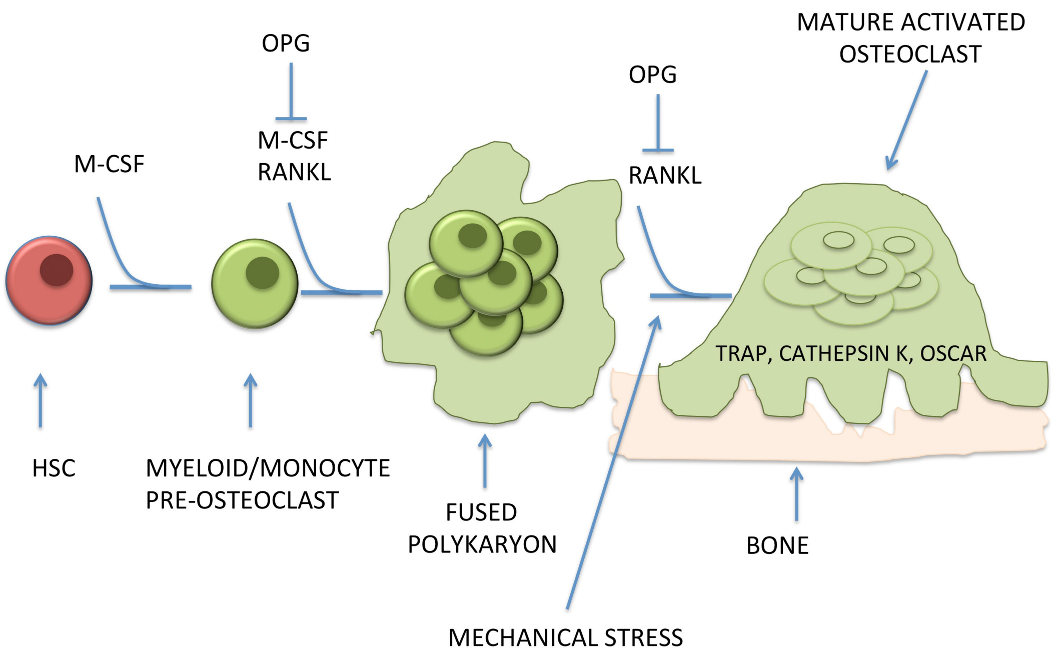

Supplementary Figure 1. *Differentiation of hemopoetic stem cells (HSC) down the osteoclastic lineage. Pre-osteoclasts fuse to form multicells or polykaryons consisting of 10 or more cells. Macrophage colony stimulating factor (M-CSF) and receptor activator of nuclear factor κB ligand (RANKL) are responsible for this process and are regulated by the soluble protein osteoprotegrin (OPG). Mechanical stress induces osteoblast stromal cells to produce RANKL, which induces the*

osteoclast maturation and activation leading to bone resorption. Mature/active osteoclasts express tartrate acid phosphatase (TRAP), cathepsin K and osteoclast associated receptor (OSCAR).

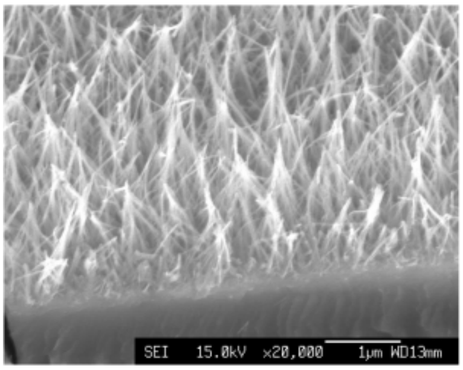

Supplementary Figure 2. *SEM images of Ti nanosurfaces. Hydrothermal treatment of titania (*TiO_2*) surfaces changed the texture of the surface from a homogeneously dense coverage of spike-like structures with an average height of 1 µm based on the scale bar provided.*

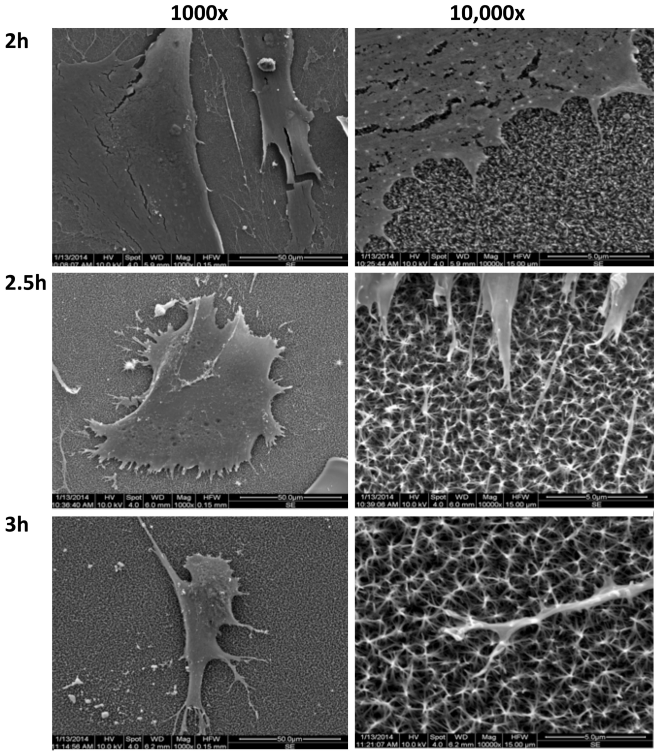

Supplementary figure 3. *SEM images of cells grown on the 2 h, 2.5 h or 3 h nanowire surfaces at 1000x magnification with shriveled appearance as the complexity increases. At 10,000x magnification it is evident that the cells extend filopodia and lamellipodia at the ridges or pockets of the nanowires.*

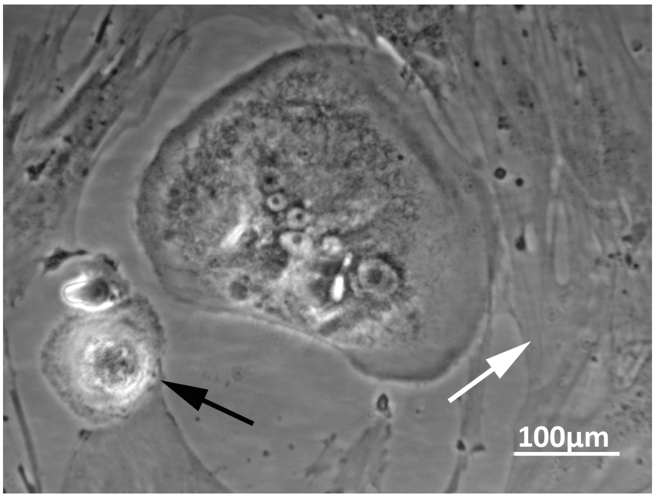

Supplementary figure 4. *Bright field microscopy of BM culture. In the middle, a large multinucleated mature osteoclast formed in tissue culture plastic after 5 week in culture. An immature osteoclast/macrophage is indicated by black arrow, surrounded by osteoblasts (white arrow). Scale bar as shown.*
